# Supplementary material for: Are carbon emissions trading and green financial instruments synergistic? -Comprehensive quantitative research based on content analysis
Source: PLoS One. 2024 Mar 7;19(3):e0298601. doi: 10.1371/journal.pone.0298601 (PMC10919601; doi:10.1371/journal.pone.0298601)
Supplement: S1 Data — (ZIP) [file pone.0298601.s001.zip › Data/地级市Z_F工作报告.zip!/░▓╗╒/░÷▓║╩╨/└┤╫╘╣1⁄2╓┌║┼í╛┬φ┐╦╩2╛▌═°í┐_2018.docx]

各位代表： 
　　 现在，我代表市人民政府向大会报告工作，请予审议，并请市政协委员和其他列席人员提出意见。 
　　 一、2017年和过去五年工作回顾 
　　 2017年，全市人民在市委的坚强领导下，认真学习宣传贯彻习近平新时代中国特色社会主义思想和党的十九大精神，全面推进淮河流域和皖北地区中心城市建设，经济社会持续健康快速发展，较好地完成了市十五届人大五次会议确定的目标任务。预计，全市实现地区生产总值1550亿元，同比增长9.3%，其中一、二、三产增加值分别增长3.5%、9.8%、10.6%；财政收入274.5亿元，增长9.3%；固定资产投资1900亿元，增长14%；社会消费品零售总额725亿元，增长12.5%；城镇、农村常住居民人均可支配收入分别达31145元、13737元，增长8.7%、9.1%；居民消费价格涨幅低于省控目标，万元地区生产总值综合能耗、主要污染物排放量持续下降。 
　　 (一)精准调度服务，经济运行稳中向好。 
　　常态化开展“四送一服”、“行走企业”活动，为企业解决8类1452个问题。打好企业降成本“组合拳”，为企业减免抵退各项税费55.8亿元，政银担在保余额52亿元，续贷转贷过桥资金、“税融通”分别放款28亿元、6亿元，兑现援企稳岗补贴7260万元。净增规上工业企业113户，规上工业企业增加值增长10%。工业经济效益综合指数400%，居全省第2位。战略性新兴产业产值占规上工业比重达35.2%，居全省第1位。中国建材高铝高碱高强盖板玻璃、盛世薄膜太阳能电池用超白基板生产线、华光高效薄膜太阳能电池高温玻璃、比亚迪云轨安徽制造中心轨道梁厂等建成投产。西部城区重点化工企业退市进园步伐加快，丰原集团VC及其衍生品、淀粉糖、L-丙氨酸、热电联产一期等项目建成投产，中粮生化燃料乙醇、天润化工聚丙烯酰胺等项目加快推进，佳先化工年产7000吨二苯甲酰甲烷、沫河口工业园热电项目开工建设，八一化工选址等前期工作全面开展。市经开区纳入国家级经开区培育名单。成功创建省新型工业化军民融合产业示范基地。国家电子商务与物流快递协同发展试点进展顺利，蚌埠电商产业园、怀远电商孵化园入围省级示范园，淮上区获批全省首家快递协同发展试验区。皖北徽商物流港入选首批省级示范物流园区。居然之家、南翔城市广场等开业运营。净增限额以上批零住餐企业40户、规上服务业企业21户。怀远县上谷农贸小镇、蚌山区国际足球小镇入选首批省级特色小镇，禹会区禾泉农创小镇、淮上区智慧物流小镇列入培育名单。大明文化园一期开园迎客，古民居博览园主体建成。旅游总收入234亿元，增长19%。设立农业产业化投资基金。启动4个食品加工产业园和3个秸秆资源综合利用环保产业园建设。新增国家级蔬菜标准园2个、省级4个。创建省级现代农业产业化联合体13家、甲级队5家。连续19年实现耕地占补平衡。粮食总产285.86万吨，实现“十四连丰”。 
　　 (二)扩大有效投入，经济发展后劲增强。龙子湖水生态环境综合治理、日丰科技电气电缆工业园等162个重点项目开工建设，豪威交互式电子白板等77个重点项目建成投产。争取中央项目补助资金9.8亿元。新增项目储备规模突破1万亿元。建立“无会周”制度，集中进行外出招商和项目推进，凯盛新型显示、鸿路绿色生态建筑产业化、南京高传机电风力发电、诚品鲜智能制造等20个10亿-100亿元项目落户蚌埠。与清华大学、中国电科、上海鹏欣集团等签署战略合作协议。实际到位市外亿元以上项目资金900亿元，增长10%。合肥科技农商行蚌埠分行正式运营。与上海久有基金公司共同设立张江高科(蚌埠)股权投资基金。安徽水利吸收合并安徽建工整体上市，雪郎生物上市辅导备案，安泰医药、中草香料等5家企业挂牌新三板。直接融资137亿元，首破百亿。与建设银行安徽省分行签署住房租赁战略合作协议。医疗服务能力提升工程获开行授信42亿元，成为国内首例特许经营权质押贷款融资案例。 
　　 (三)深化改革开放，发展活力加速释放。设立专项资金支持驻蚌单位创新发展。全社会研究与试验发展经费达31.6亿元。万人发明专利拥有量8.2件。总投资100亿元的铜铟镓硒薄膜太阳能项目国内首片高效模组成功下线。市高新区获批中国产学研创新示范基地。新认定高新技术企业51家，高新技术产业增加值增长17%。新增国家级、省级研发平台12个、众创空间4个。新引进高层次人才团队12个、省特支计划领军人才9人，新建院士工作站2个、博士后科研工作站2个。相继举办中国MEMS传感器暨集成电路产业发展(蚌埠)高峰论坛、院士安徽蚌埠行、安徽省第四届工业设计大赛暨第十二届中国工业设计周等“科技创新月”系列活动。“多证合一、一照一码”覆盖面达93%。新增登记企业8889户，增长16.1%。“皖采商城”确定为省级政府采购平台。跻身首批公立医院综合改革国家级示范城市。省级医保管理体制改革试点工作全面启动。全国商务综合执法体制改革经验获得推广。17个村开展农村集体资产股份合作制改革试点。“一户一块田”试点面积达21.6万亩。二类水运口岸获批建设。保税物流中心业务量突破4亿美元，连续两年实现翻番。实际利用外商直接投资16.1亿美元，增长7%。进出口总额18.5亿美元。蚌埠玻璃研究院、丰原集团参与“一带一路”项目建设成果丰硕。与白俄罗斯别列佐夫斯基大区签署建立友好城市关系备忘录。 
　　 (四)完善城市功能，品质能级有效提升。 
　　实施城市大建设项目218项，完成投资346亿元。《淮河流域和国家区域中心城市建设规划》、《区域性中心城市综合交通发展规划》等编制完成。G206蚌埠南段、S306淮上区段建成通车，城市云轨制造工程试验段等项目开工建设。蚌埠民用机场、水蚌线外迁前期工作取得重大进展。打通淮河路西段等断头路。开工建设各类保障性安居工程2万套。回迁安置棚改居民1.5万户。成功入选全国城市设计试点城市。新建智能立体停车场2个。新建街头绿地、游园24个，新增园林绿化395万平方米。安财商学院新校区、安徽科技学院龙湖校区、蚌埠职教园区等建成投用。义务教育均衡发展顺利通过国家复查。新建、改扩建幼儿园33所、中小学校75所，引进北大培文国际学校等优质教育资源。市三院妇儿病区、市一院妇儿辅楼交付使用，蚌医一附院肿瘤病房大楼、蚌医二附院新院区等主体完工。市体育中心、游泳跳水馆等省运会场馆基本建成，首批5个街头体育公园开工建设。市民政园主体工程封顶。舞剧《大禹》在国家大剧院成功演出。双墩、禹会村国家考古遗址公园成功立项。获批国家公共文化服务体系示范区创建城市。新增638辆新能源公交车辆，开通3条微公交线路。荣获全国公交都市示范市。 
　　 (五)深化文明创建，人居环境显著改善。 
　　坚持创建为民、创建惠民，以全国第9、全省第2的综合成绩，一举摘得全国文明城市桂冠。三县均荣获省文明县城，龙子湖区、蚌山区、淮上区获评省文明城区。整治提升586个居民小区、12个农贸市场、139条背街小巷。新建、维修公厕158座，新增停车泊位9000多个。张劼荣膺全国“时代楷模”，彭寿荣获全国道德模范提名，王广发等5人获颁“中国好人”。全面推行河长制。启动林业增绿增效行动，成片造林2.9万亩，建设农田林网76万亩，房前屋后零星植树80万株。一体化推进农村环境“三大革命”，清理陈年垃圾30万吨，建成乡镇驻地生活污水项目15个，完成改厕1万户。完成22个乡镇政府驻地建成区整治和26个省级、34个市级、38个县级美丽乡村中心村建设。固镇县禹庙村入选中国美丽乡村百佳范例。五河县荣获国家园林县城称号。龙子湖东公园生态修复项目获评中国人居环境范例奖。成功举办首届淮河流域绿色发展论坛。切实抓好中央、省环保督察整改工作，沫河口工业园区等环保突出问题得到有效解决。持续加强工业废气、城市扬尘等综合治理，淘汰黄标车3714辆，实施“明厨亮灶”工程建设1709户，中环线内9家商砼企业全部关停外迁。市生活垃圾焚烧发电、第一污水处理厂提标、怀远光大生物质发电、五河秸秆生物质发电、固镇经开区排水工程等建成投用。 
　　 (六)聚力脱贫攻坚，民生保障持续加强。 
　　始终把脱贫攻坚作为重大政治任务和最大民生工程，选派750名干部驻村帮扶、1.95万名干部对口帮扶，扎实推进九大专项行动，2.14万名贫困人口脱贫。建成“扶贫车间”141个、“扶贫基地”424个，扶持4.83万户贫困户发展产业项目5.34万个，到村产业项目实现全覆盖。投入65亿元，完成33项民生工程。城镇新增就业8.34万人。城市低保和市辖区、三县农村低保分别提标10%、19.9%、20.1%。发放救助金5.8亿元，救助各类困难对象165万人次。全面实施机关事业单位养老保险制度改革。全民国防教育、国防后备力量建设和争创全省双拥模范城市“八连冠”工作扎实推进。民族宗教、外事侨务、对台等工作不断加强，老龄、残疾人、妇女儿童、青少年等事业持续推进，公积金管理、统计、档案、地方志、社会科学、文学艺术等服务经济社会发展能力进一步增强。创新基层民政工作经验获民政部、省政府肯定推广。强化包保机制，信访总量持续下降。重点时期对重点企业采取派驻工作组等措施，安全生产形势保持稳定。积极创建国家食品安全城市。防空、防震、消防、应急等减灾防灾体系更加健全。严厉打击各类违法犯罪，坚决打击大洪山乱开滥采和淮河河道非法采砂等行为，群众安全感、满意度持续提升，获批全国首个国家级社会综合治理标准化试点市。 
　　 各位代表！ 
　　 2017年目标任务的顺利完成，为本届政府五年来的工作划上了圆满句号。过去的五年，是蚌埠改革发展进程中极不平凡的五年。我们攻坚克难，开拓进取，取得了经济社会发展的重大成就。 
　　 这五年，是坚持创新引领、争先示范，综合实力大幅提升的五年。4次荣获国家科技进步二等奖。高新技术产业增加值年均增长22.3%。地区生产总值总量前移2位，增速连续26个季度超全省平均水平。市高新区在国家高新区综合排名上升30个位次，位列第42位。成功纳入国家系统推进全面创新改革试验，合芜蚌国家自主创新示范区获国务院批准。被省委、省政府定位为淮河流域和皖北地区中心城市。 
　　 这五年，是坚持转换动能、调整结构，转型升级步伐加快的五年。二三产比重达86.7%，提高4.5个百分点。规上工业增加值年均增长11.7%，总量前移5位。战略性新兴产业产值年均增长25%，“1+3”创新产业体系初步形成。服务业增加值总量翻番。蚌埠国家级农业科技园区通过验收。获批国家级大禹文化产业示范园区。 
　　 这五年，是坚持改革攻坚、扩大开放，发展活力持续增强的五年。淮河生态经济带纳入国家、省“十三五”规划。部署重点改革任务558项，出台制度性成果118项，“一户一块田”、公立医院综合改革、公共资源交易“八统一”等改革经验在全国推介。保税物流中心、铁路无水港、蚌埠港国际集装箱码头等平台建成启用。蚌埠玻璃研究院、丰原集团等企业“走出去”成效显著。 
　　 这五年，是坚持优化环境、提升能级，城乡面貌显著变化的五年。累计实施城市大建设项目478个，投资1229亿元。改造棚户区(城中村)246个，12.8万户棚改居民实现“安居梦”。市区面积扩大355.9平方公里，建成区面积扩大38平方公里，市区常住人口增加22万人。成功创建全国文明城市、国家园林城市、国家生态文明先行示范区。 
　　 这五年，是坚持改善民生、维护稳定，群众福祉不断增进的五年。聚焦精准扶贫精准脱贫，减少贫困人口10.94万人，88个贫困村全部出列。城镇、农村常住居民人均可支配收入年均分别增长9.2%、10.7%。民生工程累计投入258.8亿元。社会保障水平持续提升，公共服务供给不断优化，社会大局保持和谐稳定。成功实现全国双拥模范城“六连冠”。 
　　 各位代表！ 
　　 五年来取得的成就来之不易，这是党中央、国务院，省委、省政府和市委坚强领导的结果，是全市人民团结奋斗的结果。我代表市人民政府，向全市人民，向人大代表、政协委员，向离退休老同志，向各民主党派、工商联、无党派人士，向各人民团体，向驻蚌解放军指战员、武警官兵和政法公安干警，向上级部门和驻蚌单位，向所有关心和支持蚌埠改革发展的各界朋友，表示崇高的敬意和衷心的感谢！ 
　　 在肯定成绩的同时，我们也深刻认识到工作中还存在不少困难和不足，主要是：发展不平衡不充分的问题还比较突出，经济总量不大与质量、效益不高并存，工业支撑、带动作用较弱，新经济、新业态、新模式还没有形成规模；创新驱动经济发展动力仍需增强；开放型经济和服务业发展短板亟需补齐；教育、医疗等公共服务与中心城市定位相比还有一定差距；县域经济发展滞缓，乡村振兴任务艰巨；大气等生态环境尚未根本好转；财政收支平衡和居民增收压力较大，防范金融风险能力有待提升，脱贫攻坚依然存在短板，民生事业仍有不少欠账；部分干部的思想作风、能力素质还不能适应新时代的要求，少数干部懒政怠政、推诿扯皮，一些领域腐败问题时有发生。对此，我们一定要高度重视，切实改进，努力把各项工作做得更好。 
　　 二、奋力开创新时代美好蚌埠现代化建设新局面 
　　 中国特色社会主义进入了新时代，我市经济已由高速增长阶段转向高质量发展阶段，正处在转变发展方式、优化经济结构、转换增长动力的攻关期，仍处于大有可为的重要战略机遇期。新时代要有新作为，我们要以永不懈怠的精神状态和一往无前的奋斗姿态，开启蚌埠现代化建设新征程。 
　　 今后五年政府工作的总体要求是：全面贯彻党的十九大精神，以习近平新时代中国特色社会主义思想为指导，坚持稳中求进工作总基调，坚持新发展理念，坚持高质量发展，坚持以供给侧结构性改革为主线，坚持以五大发展行动计划为总抓手，推动质量变革、效率变革、动力变革，坚定实施科教兴市、人才强市、创新驱动发展、乡村振兴、城乡协调发展、可持续发展、军民融合发展七大战略，坚决打好防范化解重大风险、精准脱贫、污染防治攻坚战，促进经济社会持续健康较快发展，加快建设淮河流域和皖北地区中心城市，全面建成小康社会，全面迈入全省第一方阵，奋力开创新时代美好蚌埠现代化建设新局面。 
　　 今后五年政府工作的主要任务是： 
　　 (一)奋力激活创新基因、发展潜能，建设国家创新型城市。始终把创新摆在发展全局的核心位置，用好、用活、用足系统推进全面创新改革试验、合芜蚌国家自主创新示范区等平台。以硅基和生物基为重点，依托凯盛科技集团发展壮大硅基新材料产业基地，依托丰原集团打造全产业链的聚乳酸产业园，建成双千亿新材料产业基地。依托中粮生化建成国内最大的燃料乙醇生产基地。依托中国兵器214所建设MEMS产业联盟，打造传感器产业基地。培育新能源汽车、工业机器人、高端医药等新兴产业。高新技术产业、战略性新兴产业增加值占规上工业比重分别达52%、48%以上。全社会研究与试验发展经费比重提高到3%以上，万人发明专利拥有量11件，科技进步对经济增长贡献率达60%。 
　　 (二)奋力实现质量第一、效益优先，构建现代化经济体系。认真贯彻落实习近平新时代中国特色社会主义经济思想，提高全要素生产率，加快建设实体经济、科技创新、现代金融、人力资源协同发展的产业体系。主要经济指标增速保持全省第一方阵，总量进入全省第一方阵，人均生产总值突破1万美元。聚焦工业突破，建设制造强市，规上工业企业突破2000户，争创“中国制造2025示范区”。市高新区、市经开区、蚌埠工业园区、怀远经开区、固镇经开区建成产值千亿园区，五河经开区、蚌埠(铜陵)产业园力争达到500亿。培育3-5个千亿级产业链、10个以上百亿级企业、20个以上50亿级企业。充分发展智慧经济，推动互联网、大数据、人工智能和实体经济深度融合。推动服务业突破发展，增加值占GDP比重力争达到50%。农产品加工与农业产值比达3.5。二三产业占GDP比重突破92%。 
　　 (三)奋力推进区域协调、开放合作，加快建设淮河流域和皖北地区中心城市。紧扣全国性综合交通枢纽定位，强化与周边城市互联互通，建成蚌埠民用机场，构建“米”字型快速铁路网，建设淮河中心枢纽港，形成公铁水空立体交叉的综合交通网络。积极参与“一带一路”建设，主动融入合肥都市圈、南京都市圈、长三角城市群，打造淮河生态经济带对外开放的重要战略支撑和京沪线、京福线上对外开放的重要节点城市。推进重大设施向县域延伸、重大产业向县域布局、重大项目向县域倾斜，加快振兴县域经济，实现总量占全市50%以上。着力推动基础设施、公共服务、中高端消费等快速发展，放大对皖北其他城市产业、人才、科技、金融、服务等领域的外溢效应，建设宜居宜业宜学宜游的区域性现代化大城市，打造皖北高质量发展样板区和重要经济增长极。 
　　 (四)奋力加强生态建设、环境保护，打造绿色发展蚌埠样板。牢固树立和践行绿水青山就是金山银山的理念，像对待生命一样对待生态环境。深化国家生态文明先行示范区建设，统筹山水林田湖草系统治理，实行最严格的生态环境保护制度，共建人水和谐、绿色共享的淮河生态经济带。启动大规模国土绿化行动，森林覆盖率达21%以上，建成区绿化覆盖率达45%。着力解决大气、水、土壤污染等突出问题，PM10、PM2.5平均浓度有效降低，空气质量优良率提高到80%以上，水生态环境明显好转。创建全国绿化模范城市、国家生态园林城市。 
　　 (五)奋力提升文明程度、文化自信，全面增强文化软实力。持之以恒抓好全国文明城市创建成果巩固提升工作，不断提升市民文明素质和城市文明程度。坚持干部带头，全民行动，从家庭做起，从娃娃抓起，把社会主义核心价值观融入社会发展各方面，转化为人们的情感认同和行为习惯。基本建成双墩、禹会村国家考古遗址公园，延续蚌埠历史文脉。依托国家级大禹文化产业示范园区，加快构建门类众多、特色鲜明的现代文化旅游产业体系。 
　　 (六)奋力践行共建共享、富民惠民，创造人民更加美好生活。始终坚持以人民为中心的发展思想，多谋民生之利、多解民生之忧，在幼有所育、学有所教、劳有所得、病有所医、老有所养、住有所居、弱有所扶上不断取得新进展。坚决打赢精准脱贫攻坚战，现行标准下农村贫困人口全部脱贫。就业、健康、养老、社保、住房等保障体系更加健全，教育、医疗、体育等优质公共服务水平稳步提高。弘扬生命至上、安全第一的思想，打造共建共治共享的社会治理新格局。 
　　 三、扎实做好2018年各项工作 
　　 2018年，是贯彻党的十九大精神的开局之年，是改革开放40周年，是决胜全面建成小康社会、实施“十三五”规划承上启下的关键一年，也是新一届政府的起步之年，做好今年各项工作意义重大。经济社会发展的主要预期目标是：地区生产总值增长8.6%以上；一、二、三产分别增长3.5%、9.4%和9.5%，规上工业增加值增长9.5%；财政收入增长8%以上；固定资产投资增长10.5%以上；全社会研究与试验发展经费支出增长16.5%，万人有效发明专利拥有量10件；社会消费品零售总额增长11%；进出口总额增长高于全省平均增速；城镇常住居民人均可支配收入增长与经济增长同步、高于全省平均增速，农村常住居民人均可支配收入增长高于经济增长和全省平均增速；新增城镇就业6万人以上，城镇调查失业率控制在5%以内，居民消费价格涨幅、单位生产总值能耗、国家要求削减的主要污染物排放均控制在省下达的目标以内。 
　　 重点抓好以下9个方面工作： 
　　 (一)深入实施创新驱动发展战略，加速释放发展新动能 
　　 培育转化创新成果。扎实推进“十大创新工程”。支持驻蚌科研院所创新发展，用好专项资金，推进科技成果就地转化，持续激发驻蚌科研院所、高校、企业等创新源头作用。以蚌埠玻璃研究院为龙头，打造硅基材料和新型显示创新基地，争创国家制造业创新中心。支持中国兵器214所等转制改企，加快中国电科41所系列化光纤熔接机、安瑞科星型压缩机等“军转民”、“民参军”项目建设，争创国家军民融合新型工业化示范基地。推动显示触控一体化模组、车用MEMS元器件制造及应用、5G通讯及太赫兹测试仪器等落地开工。引导支持浮法玻璃新技术等国家重点实验室提升技术攻关能力，加快实施航空装备用光电信号一体化传输模块、高硬宽谱增透型超薄光伏玻璃等省科技重大专项。继续开展“科技创新月”活动。技术合同成交金额22亿元。 
　　 壮大创新创业载体。持续深化与大院大所战略合作，加快与清华大学、中科院上硅所、深圳先研院、机械研究总院和中国电科集团、南京传动院共建创新平台。建成中科院(蚌埠)科技成果交易转化孵化中心、绿色谷创新中心，培育、支持电磁离合器、电子测试技术等技术研发平台升格为国家级。加快推进卡尔森国际科技产业园建设。新建8家省级以上研发平台。推动华东光电集成电器件研究所与清华大学组建国家高端芯片创新联盟和实体企业。新认定高新技术企业25家以上，高新技术产业增加值增长16%。支持各类创新工厂、创客空间、虚拟创新创业社区发展。科技企业孵化器面积达40万平方米，新增省级以上众创空间4个。 
　　 集聚各类创新人才。全面落实10条人才新政，建立产业领军人才信息库，力争引进国内外产业领军人才3人以上、高层次人才20人以上、产业基础人才1万人以上。新增院士工作站2个以上、博士后科研工作站3个以上。实施企业家互联网思维提升计划，造就一支具有全球视野、开拓精神、创新能力的企业家队伍。鼓励用人单位采取技术合作、技术入股、投资兴办实业等方式，柔性汇聚各类创新创业人才。在蚌埠职教园区开展“校企双制、工学一体”的现代新型学徒制试点，全面推行“订单式”人才培养。实施摇篮工程，加快建设各类中高端人才公寓、青年创业公寓、大众创业社区，吸引、留住大学生在蚌就业创业，探索建设海外人才离岸创业基地，多措并举引进人才、培养人才、用好人才。 
　　 深度优化创新生态。深化科技体制改革，建立以企业为主体、市场为导向、产学研深度融合的技术创新体系。加快科技与金融有效融合，建立从天使投资、风险投资到产业基金、上市融资的全金融产业链。谋划组建市高新区科技银行。发行中小企业科技创新券。健全知识产权创造、保护和运用体系，重拳打击侵权违法行为。大力弘扬创新文化，营造人人创新、事事创新、时时创新、处处创新的浓厚氛围。 
　　 (二)深化供给侧结构性改革，保持经济持续健康发展势头 
　　 扎实推进“破”、“立”、“降”。有效推动“僵尸企业”兼并重组、破产清算，为新产业发展腾出空间。统筹推进“三重一创”，加快建设创新型现代产业体系。突出“五大制造”，大力发展高端制造、智能制造、绿色制造、精品制造、服务型制造。培育发展智慧经济，加快宽带网络光纤化改造和三网融合，推广“智慧+”模式，实施“人工智能+”行动计划，培育新的经济增长点。加大对中小微企业的税收优惠力度，继续执行阶段性降低企业社保费率有关政策，稳步扩大电力直接交易规模，清理规范涉企保证金，大幅度压减工业产品生产许可证，大刀阔斧降低企业经营综合成本。 
　　 倾心服务实体经济。常态化、精准化开展“四送一服”、“行走企业”活动，用好“千干扶千企·千企上云端”云服务、大数据平台，健全限时反馈、限期督办、自动提醒等制度，畅通企业问题受理、解决渠道，增强企业获得感。实施重点困难企业脱困计划，建立困难企业“一企一策”帮扶清单。开展中小企业扶助专项行动，完善中小企业产业园“一站式”服务功能，新增入驻企业200家以上。实施质量品牌升级工程，积极创建全国质量强市示范城市。启动国家机械工业过滤产品质量检测中心建设。争创省著名商标25件、名牌产品12个。继续开展市长质量奖和蚌埠知名品牌评选活动。做好第四次全国经济普查工作。 
　　 不断提升投资质量。严格落实“四督四保”，突出线上线下协同调度，开工建设凯盛科技新型显示产业园等项目，加快推进泰富重装华东智能制造产业园、丰原生物产业制造基地等项目，建成蚌埠卷烟厂异地技改、诚品鲜智能制造产业园等项目，入库5000万元以上项目数突破800个，力争达到1000个。亿元以上重点项目投资超1200亿。实施重大项目谋划储备和前期工作攻坚计划，新增项目储备规模1万亿元。 
　　 (三)聚焦工业强市不动摇，打造产业升级主引擎 
　　 助推新兴产业争先领跑。强力推进硅基新材料、生物制造、智能装备制造、专用电子器件“1+3”基地建设，总产值突破2000亿元。开工年产20万吨纳米二氧化钛等项目，加快推进8.5代TFT-LCD基板玻璃、台嘉电子级玻璃纤维生产等项目，建成今上显示智能视窗保护屏和显示屏等项目。加快实施生物基新材料、通用核心器件等省级重大新兴产业工程，力争5G通信测试等省级重大新兴产业专项取得突破。支持凯盛科技、大富重工工业机器人等扩大规模。大力培育蚌埠玻璃研究院、丰原集团等一批具有创新能力的排头兵企业。战略性新兴产业产值增长20%，占规上工业比重继续保持全省前列。 
　　 加快传统产业转型升级。全面推进西部城区重点化工企业退市进园，建成投产丰原发酵苹果酸、中粮生化热电联产项目，加快建设中粮生化燃料乙醇二期、佳先化工年产7000吨二苯甲酰甲烷等项目。开工建设新源热电新厂区、丰原生物聚乳酸、八一化工硝基氯苯等项目。新增智能制造车间或生产线10个、绿色工厂4个。全面实施化工、建材、食品等传统产业扶持改造提升计划，完成技改投资500亿元，再造传统产业新优势。支持本土企业加快发展，对其新增投资项目比照招商引资政策执行。培育安徽工业精品5个、新产品20个。调度推进国电蚌埠电厂二期等“十大建成工业项目”。新增规上工业企业200户、专精特新企业30户。 
　　 促进开发区提质增效。探索开发区建设发展新模式，按照“一县一区”原则，全面完成开发区优化整合。加快补齐市政、环保、物流等短板，推广厂房代建，坚决不让项目等基础设施，坚决不让设备等厂房。全面推行开发区内企业审批、审核事项全程代办制度。支持市高新区开展创新政策试点示范。加大市经开区升格国家级培育支持力度。支持蚌埠工业园区、怀远经开区、固镇经开区扩区升级，加快沫河口高端医药产业园、临港产业园等特色园区建设。省级以上开发区规上工业增加值占全市比重达60%以上。 
　　 (四)积极培育新产业、新业态，推动现代服务业优化升级 
　　 着力提升生产性服务业。加快建设电商蚌埠，新增开展电商业务限上企业10户，网上零售额实现翻番。建成百大易商通智能物流园一期、普洛斯物流一期、合一冷链二期等项目，打造区域性物流枢纽城市。新增2家省级现代服务业集聚区。推动淮上区制造业主辅分离综合改革试点。建成投运中通蚌埠分拨中心，开工建设圆通分拨中心，快递业务量增长15%。加快曹山工业设计小镇规划建设，新增国家级、省级工业设计中心5家。 
　　 倾力优化生活性服务业。净增限上批零住餐企业20家、规上服务业企业30家。社会消费品零售总额达800亿元。建成运营义乌国际商贸城二期、绿地缤纷荟等项目，开工建设上海东鼎购物广场、汇金广场二期等项目。提升淮河路中央商务区、万达金街、银泰城第九街等特色商业街区运营水平。开展“消费总动员”活动，培育消费新模式，打造消费新热点，争创国家级信息消费试点示范市。开展旅游新型业态培育、精品路线打造、景区管理提升，推动旅游业大发展。加快农垦小镇特色景区、电影工业特区等项目建设，部分建成开放古民居博览园、白乳泉景区。启动民宿建设三年提升计划，新增星级农家乐12家、乡村旅游示范村15个、优秀旅游乡镇6个。 
　　 大力发展金融服务业。谋划建设皖北金融中心、皖北股权交易中心，引进1-2家金融机构，吸引上市公司、大型企业集团在蚌设立财务公司、租赁公司。常态化开展政银企对接活动，新增贷款210亿元。新型政银担在保余额60亿元。续贷转贷过桥资金年周转率不低于12次。新增1家企业申报上市、5家企业新三板挂牌。支持企业发行短期融资券、中期票据、集合票据等债务融资工具。直接融资100亿元。 
　　 (五)实施乡村振兴战略，加快推进农业农村现代化 
　　 加快县域工业经济发展。重点聚焦工业强县，制定县域经济发展振兴计划。设立县域工业发展基金，对产业集聚度高的工业园区、辐射带动力强的工业项目给予倾斜，推进三次产业融合发展，提升县域发展产业支撑能力。支持三县与科研院所、高等院校结对合作，推动更多创新成果在县域落地转化。鼓励金融机构在县城和乡镇设立服务网点。优化返乡创业园支持政策，突出发展创业经济。完善差异化考核评价体系，力促三县在全省考核中质量快提升、速度争先进、总量进位次、人均上台阶。 
　　 大力发展现代农业。深化农业供给侧结构性改革，走质量兴农之路。农业产业化基金规模达5亿元。加速农产品营销电商化，60%以上市级龙头企业实现线上线下同步经营。实施农业品牌战略，新认定市级知名品牌10个，新发展“三品”认证企业60家。加快建设三县、淮上区食品产业园，提高农副产品就地转化率。聚焦生物质、乳品饮料、肉制品、粮油食品、水产品5大优势产业集群，推进鹏欣大豆饲加养一体化、怀远米老头、今三麦等项目，三县各引进1个产值10亿元以上的一二三产融合项目，创建三次产业融合发展示范园。新增规模以上农产品加工企业10家，农产品加工产值960亿元，增长6.6%。粮食产量稳定在280万吨。开展玻璃智能温室建设试点。新增设施蔬菜5000亩、大水面生态养殖1.5万亩，创建3个部级、省级畜禽养殖示范场。新增市级休闲农业示范点5个。 
　　 补齐农村基础设施短板。全面推进三县、淮上区水系连通补水蓄水工程。加快淮干一般堤加固、天公河连通、怀洪新河水系洼地治理等重点水利工程建设，推进方邱湖堤防退建、天河湖平原水库等前期工作。全面加强“四好”农村公路建设。实施新一轮农村电网改造升级工程。建设高标准农田29.8万亩，发展高效节水灌溉面积2.28万亩。人工造林3.5万亩，森林抚育10万亩，建设农田林网示范片10个，村庄房前屋后零星植树100万株，提升绿化品质、结构和质量。扎实推进农村环境“三大革命”和农村安全饮水全覆盖，全面改善农村人居环境。农村生活垃圾无害化处理率55%。完成33个、新启动5个乡镇污水处理设施项目。完成8万户农村厕所改造。完成乡镇政府驻地建成区整治和美丽乡村中心村建设任务。持续推进市区优质公共服务资源向县域下沉，不断提升教育、医疗、文化等服务水平。 
　　 稳步推进农村综合改革。巩固和完善农村基本经营制度，深化农村土地制度改革，完善承包地“三权”分置制度。开展土地入股、托管试点。新增新型农业经营主体500家、服务主体100家。有序推进农村集体产权制度改革，基本完成乡镇清产核资工作，完成农村股份合作制改革达20%。扩大“资源变资产、资金变股金、农民变股东”试点。完成20个村的“村改居”工作。加强农村基层基础工作，健全自治、法治、德治相结合的乡村治理体系。培养造就一支懂农业、爱农村、爱农民的“三农”工作队伍。 
　　 (六)完善承载服务功能，提升中心城市品质能级 
　　 增强综合交通枢纽功能。加快实施蚌五高速公路、蚌固高速公路、G329蚌埠至怀远段、水蚌线外迁等项目，建成城市云轨制造工程试验段、京台高速东海大道出入口。开工建设刘府高速出口连接线和中环线西北环、西南环。做好G345马城至唐集段、G206怀远至宿州段等项目工作。加快淮河航道等级提升、涡河航道治理，推进长淮卫港口作业区、五河城南、固镇浍河新港码头等港口群建设。积极推进蚌埠南站扩建、沿淮铁路、皖北城际铁路、动车运用所、城市云轨等前期工作。力争开工建设蚌埠民用机场、合新高铁五河段等项目。 
　　 系统推进城市更新。以居民需求为导向，强化市政基础、公共安全等设施规划建设和管控，补齐功能短板。实施城市大建设项目180个，完成投资320亿元。启动高铁新区开发建设和西部城区企业退市进园区域规划建设。深入推进“两治三改”，治理违法建设14万平方米、违法用地291亩，实施棚户区(城中村)改造1.23万户、老旧小区改造78个。打通大庆路北段等断头路，完成朝阳路等主次干道“白改黑”。加快建设司马庄路等3条地下综合管廊和大禹家园等海绵城市工程。新建立体停车场5处，新增机动车停车泊位4.5万个。 
　　 稳妥推行住房制度改革。按照“房子是用来住的、不是用来炒的”定位，积极探索创新，加快建立多主体供应、多渠道保障、租购并举的住房制度。加大公租房保障力度，优先保障环卫、公交等行业困难群体，将符合条件的新就业无房职工、外来务工人员和青年学生、青年教师等纳入保障范围，对低保、低收入住房困难家庭实现应保尽保。加强住房市场监管，保持房地产调控政策连续性和稳定性，避免房价大起大落，完善房地产市场平稳健康发展长效机制。 
　　 提升公共服务供给水平。坚持教育优先发展，深入推进义务教育城乡一体化和优质均衡发展，普及高中教育，着力解决中小学生课外负担重、“择校热”、“大班额”和学龄前儿童“入托难”等问题。建成北大培文学校、行知实验学校新校区、二中禹会实验学校，加快建设中科院实验学校、淮滨实验学校等。 
　　推进健康蚌埠建设，全方位、全周期保障人民健康。建成医养护理院、蚌医二附院新院区，推进市三院改扩建工程、市心血管病专科医院、皖北妇幼保健院、市一院妇产儿童医院建设，启动皖北中心医院前期工作。建成蚌埠民政园、淮上中科生态养老示范园一期等项目，发展以社区为依托的居家养老，创建国家医养结合试点市。建设涂山国家级登山步道、10个街头体育公园，确保市体育中心等省运会场馆如期建成，强化运动员备战训练，当好东道主，办好省运会。 
　　 (七)深化改革扩大开放，持续激发发展潜力活力 
　　 深入推进改革攻坚。深化“放管服”综合改革，启动实施市场准入负面清单制度，完善权力、责任、公共服务、行政权力中介服务清单动态调整机制。深化户籍制度改革，推进基本公共服务向常住人口全覆盖，提高户籍人口城镇化率，真正让农民“进得来、留得住、生活好”。稳步推进市区财政事权和支出责任划分改革。深化事业单位改革，推进政事分开、事企分开、管办分离。完成省医保管理体制改革、食用农产品产地准出和市场准入等试点任务，形成成熟模式和成功经验。统筹推进创业就业、社会治理等领域综合改革。 
　　 激发民营经济发展活力。激发和保护企业家精神，鼓励更多社会主体投身创新创业。落实支持民营企业和中小微企业的各项政策措施，破除各类歧视性限制和隐性障碍，构建亲清新型政商关系。加大基础设施、社会事业和公用事业等领域开放力度，完善PPP项目价格和收费项目适时调整机制，开展民间投资项目报建审批情况清理核查和政府失信行为专项整治，激发民间投资活力。 
　　 掀起招商引资热潮。突出产业链招商、以商招商，积极推行专业化招商、联合基金招商等新模式，引进市外亿元以上项目200个以上，到位资金1000亿元。每个县区引进2个20亿元以上产业类项目。聚焦长三角、珠三角、京津冀等重点区域，着力引进创思特光电、国轩集团等“1+3”创新产业行业龙头企业和关键配套企业，全面承接大富重工等企业搬迁转移落户蚌埠。强化招商引资咨询、论证、评估、服务等全过程管理，推进绿色可持续招商，全力塑造蚌埠最佳投资目的地品牌形象。 
　　 加快对外开放步伐。建成二类水运口岸、进境粮食指定口岸，申建进口肉类指定查验场，开辟外贸集装箱班轮航线，打通铁海联运东向、南向入海通道。保税物流中心报关总额突破6亿美元。壮大国家科技兴贸出口创新等基地，新增实绩外贸企业50家。加快“走出去”步伐，支持蚌埠玻璃研究院、丰原集团等在境外建设经贸合作园区。 
　　 (八)坚决打赢三大攻坚战，决胜全面建成小康社会 
　　 坚决打好防范化解重大风险攻坚战。建立金融风险评估会商制度和联合处置机制，确保所有金融活动监管全覆盖、无死角。开展非法违规金融活动打击行动，重点整治乱办金融、非法集资、乱加杠杆等严重干扰金融市场秩序行为；开展互联网金融专项整治行动，有序推进P2P网络借贷、股权众筹、第三方支付等领域专项整治工作；开展地方金融机构不良资产降压行动，坚决守住不发生区域性系统性金融风险的底线。稳妥处置政府债务风险，组织开展“四清四实”专项整治，着力解决政府隐性债务问题。规范政府举债行为，常态化加强全口径政府举债管理，加快政府平台公司市场化转型步伐，加强对融资平台、产业投资基金、PPP项目、政府购买服务的动态监管，强化财政预算硬约束，严控隐性债务增量。严格实行政府举债终身问责制和债务问题倒查机制，做到守土有责、守土尽责。 
　　 坚决打好精准脱贫攻坚战。坚持把脱贫攻坚作为全面建成小康社会的重中之重，全力推进九大专项行动，确保怀远县如期摘帽，全市再减少贫困人口1.4万人。加大财政存量资金扶贫投入，可统筹使用部分50%以上用于脱贫攻坚。聚焦产业扶贫，推行“四带一自”模式，建设扶贫车间50个、“扶贫基地”60个；贫困人口劳动技能培训1000人次以上，帮扶贫困劳动者就业2000人以上；每个贫困村培养1名电商扶贫能手；打造3个省乡村旅游重点村。推进贫困人口“三保障一兜底一补充”综合医保政策落实、一站式结算、大病慢性病分类救治，因病致贫、因病返贫率下降3个百分点以上。集中攻坚行蓄洪区和100个薄弱村，加大资金投入和政策支持力度。更加注重扶贫同扶志、扶智相结合，保证现行标准下的脱贫质量，切实做到 “脱真贫、真脱贫”。 
　　 坚决打好污染防治攻坚战。持续抓好中央、省环保督察反馈问题整改。完成第二次全国污染源普查工作。打赢蓝天保卫战，完成建成区餐饮油烟专项整治，开展人工影响天气改善空气质量省试点。继续实施全年全域全面秸秆禁烧，拓展秸秆综合利用，争取怀远龙亢秸秆资源综合利用环保产业园获批省级产业园，加快五河、固镇市级秸秆资源综合利用环保产业园建设，秸秆综合利用率90%，资源化利用率27.5%。严格落实林长制，推进曹山森林公园、生态植物园等项目建设。PM2.5平均浓度降到57微克/立方米，城市空气质量全年优良天数达75%以上。打好碧水攻坚战，严格落实河长制，启动实施湖长制。开展集中养殖粪污资源化利用行动，利用率达70%以上。强力实施开发区环保基础设施攻坚年行动，加快沫河口园区污水处理厂、固镇经开区集中供热二期、怀远龙亢经开区污水处理厂等建设。开展城乡黑臭水体治理行动，完成八里沟、迎河、四十米大沟等黑臭水体治理，加快鲍家沟、北淝河下游等水系治理。国家考核的水质优良断面比例达75%以上。加强沱湖、三汊河等自然保护区、湿地生态功能区保护与管理。实施饮用水水源地环境保护工程。打好净土持久战，以农用地和重点行业企业用地为重点，开展土壤污染状况详查，抓好污染土地的管控和安全利用，加大固体废弃物处理和农业面源污染防治力度，保障农产品质量和人居环境安全。开工建设餐厨废弃物处理中心、污泥深度处理中心等项目，着力打造静脉产业园。 
　　 (九)切实办好民生实事，回应群众对美好生活的新期待 
　　 统筹推进社会建设。全面完成33项民生工程，扎实推进“十大富民举措”。坚持就业优先战略和积极就业政策，开展就业创业“四进四扶”活动，统筹抓好农村转移劳动力、退役军人等各类群体就业工作。全面实施全民参保计划。加大对城乡低保户、五保户、孤儿、重症患者等困难群体的救助力度，多形式、多渠道开展慈善救助活动。加强计划生育服务管理。新增、优化公交线路8条，新增新能源公交车200台。实施全民科学素质行动计划。加强新形势下全民国防教育、国防动员、人民防空和双拥优抚安置工作，巩固和促进军政军民团结。统筹抓好民族宗教、气象、防震、应急管理、外事侨务、档案、地方志、公积金管理、统计、审计等工作，加快老龄、残疾人、妇女儿童、青少年等事业发展。 
　　 巩固提升文明创建成果。培育和践行社会主义核心价值观，传承和弘扬中华优秀传统文化。持续开展交通秩序等“十项提升行动”，推动文明创建向县、乡、村拓展。加强烟花爆竹燃放、犬只管理，倡导文明祭祀。推进诚信建设和志愿服务制度化，持续开展“诚信蚌埠·孝善珠城”等系列活动，培厚好人之城根基。建设20座书屋、20个阅读点，倡导全民阅读，共建书香蚌埠。启动全国绿化模范城市、国家生态园林城市、国家公共文化服务体系示范区创建工作。 
　　 推进社会治理创新。严格落实重大决策社会稳定风险评估制度和重大不稳定问题清单制度。加快推进“雪亮工程”建设，完善立体化、信息化社会治安防控体系，争创全国社会治安综合治理优秀城市。落实“七五”普法规划。加强法律服务和法律援助。强化信访稳定工作，完善矛盾纠纷多元化解机制。突出抓好道路交通、消防、危化品等重点领域安全生产。开展安全保障监测体系建设行动、地质灾害防御行动。加强食品药品安全监管，全面推进国家食品安全城市创建工作。依法严厉打击各类刑事犯罪，坚决维护社会大局和谐稳定。 
　　四、全面加强政府自身建设 
　　 过去的一年，我们扎实推进“两学一做”学习教育常态化制度化，组织开展“讲政治、重规矩、作表率”专题教育、警示教育。严格落实重点工作、重点项目“一周一分析调度、一月一督查推进”制度。建成运营电子政务移动办公系统APP，推进政府工作台账电子化。“放管服”综合改革深入推进，“互联网+政务服务”纳入全省试点，率先实现市、县、乡镇、村四级网上政务服务平台全覆盖，92.4%的市本级审批实现“不见面”或“最多跑一次”，96.5%的公共服务事项依申请办结。市长热线通过省级标准化试点验收。自觉接受人大监督和政协民主监督，办理市人大代表议案8件、建议155件，市政协提案491件。落实省委巡视整改任务，推进依法行政，强化监察和审计工作，完善建章立制，营造了风清气正、干事创业的良好政治生态。 
　　 打铁必须自身硬。我们将牢固树立“四个意识”，坚决维护党中央权威和集中统一领导，坚决忠诚维护看齐习近平总书记这个党的领袖，坚决维护习近平总书记在党中央和全党的核心地位。按照“既要政治过硬，也要本领高强”的要求，自觉用习近平新时代中国特色社会主义思想武装头脑，深入学习宣传贯彻党的十九大精神，认真开展“不忘初心、牢记使命”主题教育，全面加强政府系统自身建设。 
　　 (一)依法行政，塑造阳光法治的政府形象。始终把依法行政作为工作准则，坚持公正为民、严格执法。落实重大行政决策法定程序，开展重大决策第三方评估，广泛征求群众意见，把政府决策过程变为汇民意、聚众智、凝共识的过程。自觉接受党内监督、人大监督、政协民主监督。认真执行市人大及其常委会的决议和决定。切实办好人大代表议案、建议和政协提案，全面实行领导干部领办机制。对议案、重点建议和重点提案，实行市政府分管领导领办；各承办单位办理的所有答复要召集会议集体研究，并报经市政府分管领导同意后答复。虚心听取各民主党派、工商联、无党派人士意见，充分发挥工会、共青团、妇联等人民团体桥梁纽带作用。高度重视司法监督、群众监督、舆论监督，让行政权力始终在法治轨道上阳光运行。 
　 (二)牢记宗旨，践行以人民为中心的发展思想。始终把人民利益摆在至高无上的地位，关注民生的态度坚定不移、改善民生的力度只增不减、保障民生的投入只增不减。大兴调查研究之风，推行一线工作法，更多运用“四不两直”、现场办公等方式，推动干部在一线干事，问题在一线解决。健全政务公开、政府信息发布和网络诉求跟踪办理等制度，提升市长热线、政行风热线办理质量，及时回应社会关切。 
　　 (三)勇于革命，创优“四最”的营商环境。 
　　放大商事制度改革效应，统筹推进“多证合一”、“证照分离”、“先照后证”、“照后减证”改革，做到审批事项全省最少。开展政务服务便民行动，完善“互联网+政务服务”体系，大力推进一网通办和“不见面审批”，做到办事效率全省最高。对企业推行区域评价、多评合一，把市场能有效调节的交给市场，企业能自主决策的还给企业，做到投资环境全省最优。坚决杜绝少数单位“门好进、脸好看”就是“事难办”等“四风”新问题，当好服务群众、服务企业、服务基层的“店小二”，做到市场主体和人民群众获得感全省最强。 
　　 (四)抓铁有痕，形成务实高效的行政作风。 
　　始终把抓落实、提效率作为政府工作的主旋律，对每项工作实行任务菜单式分解、责任契约式落实、绩效审计式验收、结果问责式考核，对重点工作、重大项目坚持“一周一分析调度、一月一督查推进”。完善正向激励和容错纠错机制，旗帜鲜明地为敢于担当的干部担当，为敢于负责的干部负责。坚决破除老办法不管用、新办法不会用的掣肘，各项工作在全国争试点、全省争先进、区域争一流，为蚌埠跨越式发展努力到殚精竭虑、奋斗到感动自己！ 
　　 (五)廉洁从政，维护山清水秀的政治生态。认真履行“一岗双责”，强化“两个责任”，加强日常监督和审计监督，抓好巡视巡察和审计问题的整改，铲除滋生腐败的土壤。坚决向行政执法中的乱检查、乱收费、乱罚款亮剑，坚决向工程建设中的强揽工程、强买强卖亮剑，坚决向办事服务中的索拿卡要、设租寻租、不作为慢作为乱作为亮剑，坚决向公共资源交易和政府性投资项目招投标中的暗箱操作、串标围标、“猫鼠同眠”亮剑，坚决向侵害群众利益的蚁腐村霸亮剑，让破坏发展环境、损害人民利益的人和事在蚌埠无藏身之处、无立锥之地！ 
　　 各位代表！ 
　　 新时代赋予新使命，新作为开启新征程。让我们更加紧密地团结在以习近平同志为核心的党中央周围，在省委、省政府和市委的坚强领导下，不忘初心，牢记使命，撸起袖子加油干，大干苦干加实干，为建设淮河流域和皖北地区中心城市，全面建成小康社会、全面迈入全省第一方阵，奋力开创新时代美好蚌埠现代化建设新局面而努力奋斗！
